# Risk prediction models for colorectal cancer in people with symptoms: a systematic review

**DOI:** 10.1186/s12876-016-0475-7

**Published:** 2016-06-13

**Authors:** Tom G. S. Williams, Joaquín Cubiella, Simon J. Griffin, Fiona M. Walter, Juliet A. Usher-Smith

**Affiliations:** School of Clinical Medicine, University of Cambridge, Cambridge, UK; Department of Gastroenterology, Complexo Hospitalario Universitario de Ourense, Instituto de Investigación Biomédica Ourense-Vigo-Pontevedra, Ourense, Spain; The Primary Care Unit, Department of Public Health and Primary Care, University of Cambridge, Cambridge, CB1 8RN UK

**Keywords:** Colorectal cancer, Risk, Model, Symptoms, Prediction

## Abstract

**Background:**

Colorectal cancer (CRC) is the fourth leading cause of cancer-related death in Europe and the United States. Detecting the disease at an early stage improves outcomes. Risk prediction models which combine multiple risk factors and symptoms have the potential to improve timely diagnosis. The aim of this review is to systematically identify and compare the performance of models that predict the risk of primary CRC among symptomatic individuals.

**Methods:**

We searched Medline and EMBASE to identify primary research studies reporting, validating or assessing the impact of models. For inclusion, models needed to assess a combination of risk factors that included symptoms, present data on model performance, and be applicable to the general population. Screening of studies for inclusion and data extraction were completed independently by at least two researchers.

**Results:**

Twelve thousand eight hundred eight papers were identified from the literature search and three through citation searching. 18 papers describing 15 risk models were included. Nine were developed in primary care populations and six in secondary care. Four had good discrimination (AUROC > 0.8) in external validation studies, and sensitivity and specificity ranged from 0.25 and 0.99 to 0.99 and 0.46 depending on the cut-off chosen.

**Conclusions:**

Models with good discrimination have been developed in both primary and secondary care populations. Most contain variables that are easily obtainable in a single consultation, but further research is needed to assess clinical utility before they are incorporated into practice.

**Electronic supplementary material:**

The online version of this article (doi:10.1186/s12876-016-0475-7) contains supplementary material, which is available to authorized users.

## Background

Colorectal cancer (CRC) is the third most common cancer worldwide and the fourth leading cause of cancer-related death [1]. Detecting the disease at an early stage improves outcomes [2]. Whilst screening has been successful in reducing the incidence and mortality of CRC by increasing the proportion diagnosed at an early stage and facilitating removal of pre-neoplastic lesions [3–5], the majority of cancers are still diagnosed after symptomatic presentation [6]. Three previous meta-analyses have shown that individual symptoms, such as rectal bleeding and change in bowel habit, are associated with CRC, but are also common in populations without cancer and so have poor sensitivity for CRC [7–9]. Consequently, identifying which patients from primary care should be referred for diagnostic investigation remains challenging.

Several approaches have been developed to improve the appropriateness of referrals for investigation of symptoms suggestive of CRC and reduce delays in diagnosis. The NHS in England introduced the two-week wait (2WW) referral system in 2000, followed by the NICE suspected cancer referral guidelines in 2005 which have been recently updated [10]. A number of evaluations have shown that the 2WW referral system for suspected CRC has variable sensitivity and low specificity and does not improve diagnostic accuracy [9, 11–16]. In recent years, several predictive models have been developed to identify people at higher risk of CRC among those with symptoms. These have the potential to improve the consistency and quality of clinical decision-making. However, their strengths, weaknesses and relative performance are uncertain, and few direct comparisons have been made. The aim of this review was to systematically identify and compare the performance of models that predict the risk of undiagnosed prevalent primary CRC for symptomatic individuals.

## Methods

We performed a systematic literature review following an a priori established study protocol (available on request) that followed the PRISMA guidelines (see Additional file 1 for the PRISMA checklist).

### Search strategy

We used a combination of subject headings including ‘colorectal cancer’, ‘risk/risk factor/risk assessment/chance’ and ‘prediction/model/score’ to conduct an electronic literature search within Medline and EMBASE. The search period ran from January 2000 to March 2014 (see Additional file 2 for the complete search strategy for Medline and EMBASE). We subsequently hand searched the reference lists of all included papers. We also considered for inclusion papers published before 2000 describing the development of models that were validated in included papers.

### Study selection

To be included, studies had to be published as a primary research paper in a peer-reviewed journal and either describe, validate or assess the impact of a risk model that allowed identification of people at higher risk of CRC or CRC and advanced colorectal neoplasia. The risk model had to feature two or more risk factors, including symptoms, for prevalent undiagnosed colorectal cancer at the level of the individual. In addition, a quantitative measure of model performance was required. Conference proceedings, papers not in English, and studies of a specific patient group, for example immunosuppressed patients or patients with a past history of CRC, were excluded.

One reviewer (JUS) screened the titles and abstracts of papers identified by the Medline and EMBASE searches to exclude studies that were clearly not relevant. A second reviewer (TGSW) independently assessed a random selection of 10 % of the papers at title and abstract level and both reviewers (TGSW and JUS) independently assessed all the full text of papers if a definite decision to reject could not be made based on title and abstract alone. All reviewers met to discuss discrepancies and reach a consensus decision on inclusion or exclusion.

### Data extraction and synthesis

Two reviewers (TGSW and JC) extracted data from each paper using a standardised form. Discrepancies were examined and resolved by a third reviewer (JUS). We extracted information on the components of each risk model and potential sources of bias. These included: study design and participants; methods of model development; and variables included in the risk model. The methods of studies published for each risk model was also classified according to the TRIPOD guidelines (1a-Development only; 1b-Development and validation using resampling; 2a-Random split-sample development and validation; 2b-Non-random split-sample development and validation; 3-Development and validation using separate data; 4-Validation study) [17]. Where multiple models were described within the same study, each model was included separately.

Reported measures of discrimination (area under the receiver operating characteristic curve (AUROC)), accuracy (sensitivity and specificity), calibration and utility were used to compare the performance of different risk models and thresholds in development and validation populations. Numerical values for the AUROC were used to compare discrimination and the sensitivity and specificity to compare the accuracy, of different models and thresholds. For those papers in which sensitivity and specificity were not reported explicitly, where possible we calculated the values from data provided in the paper. Figures were produced using RevMan version 5.3 and where multiple studies reported the sensitivity and specificity of the same model at the same threshold, the combined values were calculated using Meta-DiSc version 1.4.

### Quality assessment

Quality assessment was performed at the same time as data extraction. Since our review included studies with different designs we used a checklist based on the Critical Appraisal Skills Programme guidelines for case-control and cohort studies [18] as an initial framework, and then classified each study as high, medium or low quality. No studies were excluded based on quality assessment alone.

## Results

### Included studies

**Fig. 1:**
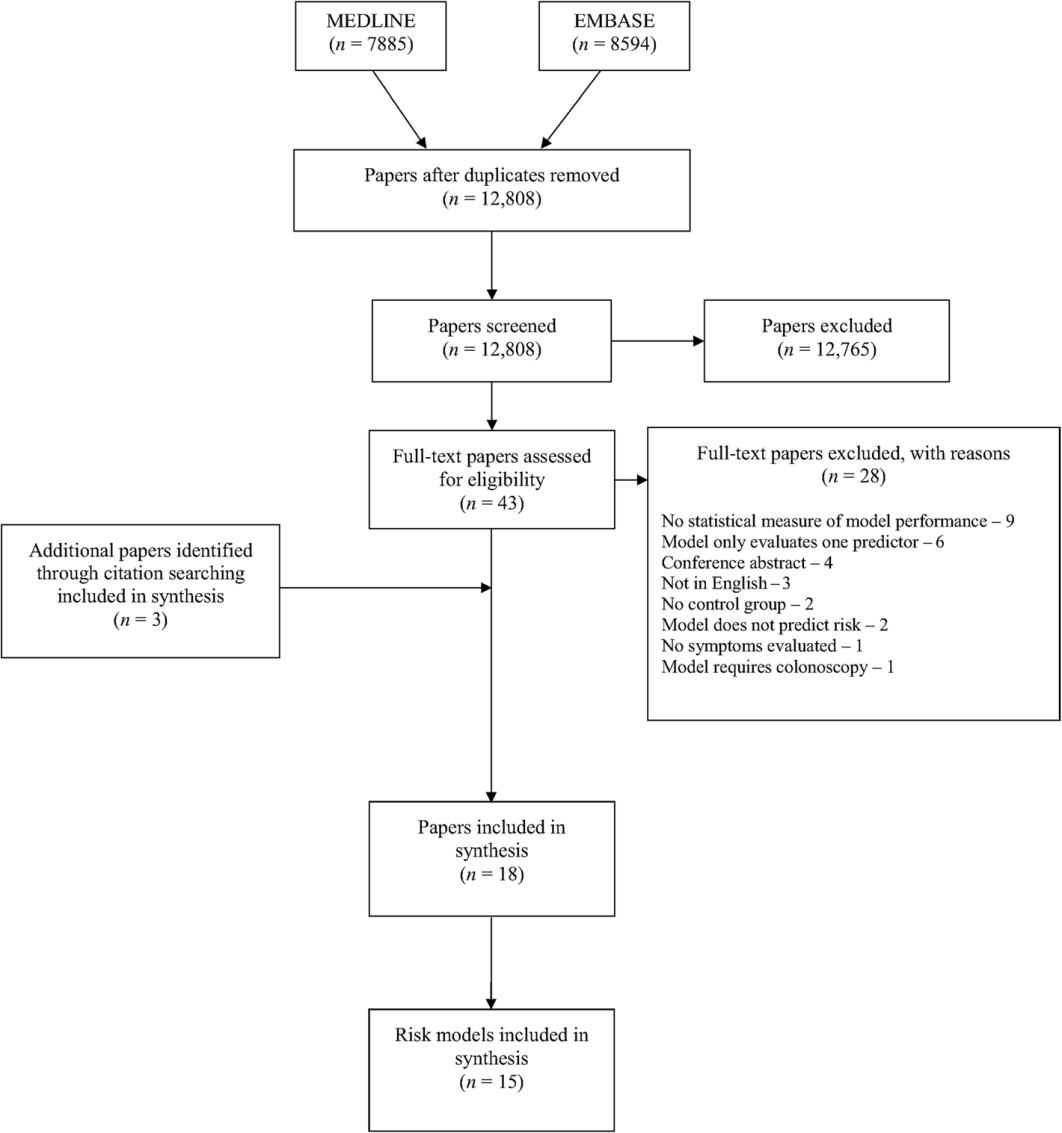
PRISMA flow diagram

After duplicates were removed, the search identified 12,808 papers of which 12,765 were excluded at title and abstract level. A further 29 were excluded after full-text assessment by at least two reviewers (TGSW and JUS). There was complete agreement among researchers throughout the screening process. The most common reasons for exclusion were that the papers did not report a statistical measure of model performance (*n* = 9), only evaluated one predictor (*n* = 6), or were conference abstracts (*n* = 4). Three additional papers were identified through citation searching, including one published prior to 2000 which was included as it had been externally validated in one of the papers identified through the literature search. In total we included 18 papers describing 15 risk prediction models in the review (Fig. 1). Only one paper assessed the impact of one of the models in practice [19].

A summary of the quality assessment of each of the 18 papers is given in Table 1. Eleven were assessed as high quality, four as medium quality and three as low quality. The studies assessed as low quality were two cross-sectional studies recruiting patients presenting to either primary or secondary care with rectal bleeding [20, 21], and the single study assessing model impact [19].

**Table 1:** Summary of quality assessment and study design for the 18 included papers

Author, date, country, setting	Quality^f^	Outcome	Data collection	Selection of variables	Identification of study population		Identification of outcome cases	Exclusions	Study population	
Model development; Case-control studies										
					Cases	Controls			Cases	Controls
ᅟHamilton, 2005, UK, primary care [22]	M	CRC	Primary care records from 21 practices	Occurring in at least 2.5 % of cases or controls	>40 years with primary CRC	5 controls per case matched for sex, general practice and age and alive at point of case diagnosis	Cancer registry at one hospital	Unobtainable records, no consultations in 2 years before diagnosis, previous CRC, residence outside Exeter at time of diagnosis.	349	1,744
ᅟHamilton, 2009, UK, primary care [23]	M	CRC	THIN database	Literature review	> 30 years with CRC	Up to 7 controls without CRC matched for practice, sex and age	Diagnosis of CRC within study period	< 2 years of full electronic records before date of case diagnosis.	5,477	38,314
Model development and external validation; Case-control study										
					Cases	Controls			Cases	Controls
ᅟMarshall, 2011, UK, primary care [24]	H	CRC	BB equation development and CAPER Score external validation							
			See Hamilton, 2009 [23]					As in Hamilton, 2009 plus patients with severe anaemia (Hb < 10 g/dl), rectal bleeding, abnormal rectal examination or positive FOBT, or without any of abdominal pain, weight loss, diarrhoea or constipation	117	433
		CRC	CAPER Score development and BB equation external validation							
			See Hamilton, 2005 [22]							
Model development and random split-sample internal validation; Cohort studies										
									Included	Cases (% of included)
ᅟHippisley-Cox, 2012, UK, primary care ᅟ(QCancer® (colon)) [25]	H	CRC	QResearch database	'Established predictor variables' and red flag symptoms	30–84 year-old patients registered with practices between 1/1/2000 and 30/09/2010 and without CRC		Incident cancer diagnosis in the 2 years after cohort entry recorded in GP records or ONS cause-of-death record	History of CRC, recorded red flag symptom^f^ in the 12 months preceding the study date, or missing Townsend deprivation score.	Development	
									F: 1,172,670	F:4,798 (0.2 %)
									M:1,178,382	M:4,798 (0.2 %)
									Internal validation	
									F: 616,361	F: 2603 (0.2 %)
									M: 620,240	M:2603 (0.2 %)
ᅟHippisley-Cox, 2013 (female), UK, primary care ᅟ(QCancer® (combined)) [26]	H	CRC and 11 other cancers^a^	QResearch database	Previous study, and literature review	25–89 year-old patients registered with practices between 1/1/2000 and 1/04/2012 and without CRC		Incident cancer diagnosis in the 2 years after cohort entry recorded in GP records or ONS cause-of-death record	Recorded red flag symptom^f^ in the 12 months before the study entry date, or missing Townsend deprivation score.	Development	
									1,240,864	2607 (0.18 %)
									Internal validation	
									679,174	1725 (0.25 %)
ᅟHippisley-Cox, 2013 (male), UK, primary care ᅟ(QCancer® (combined)) [27]	H	CRC and 9 other cancers^b^	QResearch database	Previous study, and literature review	25–89 year-old patients registered with practices between 1/1/2000 and 1/04/2012 and without CRC		Incident cancer diagnosis in the 2 years after cohort entry recorded in GP records or ONS cause-of-death record	Recorded red flag symptom^f^ in the 12 months before the study entry date, or missing Townsend deprivation score.	Development	
									1,263,071	3250 (0.26 %)
									Internal validation	
									667,603	1356 (0.2 %)
Model development; Cross-sectional studies										
									Included	Cases (% of included)
ᅟAdelstein, 2010, Australia, secondary care [32]	H	CRC	Self-administered questionnaire	Not reported	Patients > 18 years old scheduled for colonoscopy at hospitals		Complete colonoscopy and histology	Completion of questionnaire > 6 months before colonoscopy, advanced adenoma^c^, incomplete colon evaluation	7,736	159 (2.1 %)
ᅟAdelstein, 2011, Australia, secondary care [31]	H	CRC	See Adelstein 2010 [32]					Completion of questionnaire > 6 months before colonoscopy, adenoma^d^, incomplete colon evaluation	6943	159 (2.3 %)
ᅟFijten, 1995, Netherlands, primary care [21]	L	CRC	Patient and doctor questionnaires, and blood sample	Literature review	Patients presenting to 83 GP practices with overt rectal bleeding or a history of visible rectal blood loss in previous 3 months.		Medical record review coded using the International Classification of Primary Care for diagnostic classification	Patients aged <18 or >75, pregnancy, urgent admission to hospital or follow-up not available.	290	9 (3.4 %)
ᅟHurst, 2007, UK, secondary care [28]	M	CRC or pre-malignant adenomas	Proforma-based history, examination and blood sample	Not reported	All adult patients referred to a specialist colorectal clinic		Patients tracked until a definitive diagnosis was reached	Patients not further investigated after initial consultation or who did not attend follow up	300	95 (31.7 %)
ᅟLam, 2002, Hong Kong, secondary care [20]	L	CRC or significant neoplasia^e^	Questionnaire conducted by non-medically trained interviewers	Not reported	New patients attending surgical department for rectal bleeding		Rigid sigmoidoscopy and proctoscopy, followed by barium enema or colonoscopy at the physician's discretion	Refusal for colonoscopy or barium enema	174	29 (16.7 %)
ᅟMahadavan, 2011, UK, secondary care [29]	M	CRC	Self-administered questionnaire, history, faecal, blood and rectal samples	Not reported	All patients >40 years referred to a surgical clinic via the 2ww^g^ system for colorectal cancer		Incident diagnosis of CRC within 6 months of study entry from primary care or hospital records confirmed histologically	Previous confirmed IBD, GI cancer, investigation of the bowel within the last 6 months or absent rectal sampling result	714	72 (10.1 %)
Model development and external validation; Cross-sectional study										
									Included	Cases (% of included)
ᅟSelvachandran, 2002, UK, secondary care (WNS) [30] ^h^	H	CRC	Self-administered questionnaire	Not reported	Patients referred by GPs with symptoms suggestive of distal colonic or anorectal disease		Not reported (all patient's received endoscopy)	Not reported	2,268	95 (4.2 %)
Model external validation; Cohort study										
		Model(s) validated							Included	Cases (% of included)
ᅟCollins, 2012, UK, primary care [33]	H	QCancer® (colon) (female and male) [25]	THIN database	N/A	30–84 year-old patients registered with practices between 1/1/2000 and 30/09/2010 and without CRC		Incident cancer diagnosis of CRC in the 2 years after cohort entry	Patients with a history of CRC, a recorded red flag symptom^f^ in the 12 months preceding the study date, registered <12 months with practice or with invalid dates	Female: 1,075,775	Female:1,676 (0.15 %)
									Male: 1,059,765	Male: 2,036 (0.19 %)
Model external validation; Cross-sectional studies										
		Model(s) Validated							Included (% of eligible)	Cases (% of included)
ᅟBallal, 2009, UK, secondary care [35]	H	WNS [30]	Self-administered questionnaire	N/A	Patients with colorectal symptoms referred by GPs		A combination of rigid sigmoidoscopy, flexible sigmoidoscopy, colonoscopy or barium enema	Patients thought (on the basis of the referral) most likely to have right-sided CRC, or but did not attend for investigation	3,457	186 (5.4 %)
ᅟHodder, 2005, UK, secondary care [34]	H	WNS [30], Fijten 1995 [21]	Self-administered questionnaire	N/A	Patients referred from primary care with colorectal symptoms		Secondary care investigations - minimum flexible sigmoidoscopy	Not reported	3,302	156 (4.7 %)
ᅟRai, 2008, UK, secondary care [11]	H	WNS [30]	Self-administered questionnaire	N/A	GP referral with any of: lower bowel-related symptoms, unexplained iron deficiency anaemia, positive FOBT, or palpable rectal/abdominal mass		Follow up during course of hospital investigations until a final diagnosis made	Patients admitted hospital as an emergency and subsequently diagnosed with CRC	1,422	83 (5.84 %)
Model utility; cohort study										
		Model used					Outcome measures		Included	Interviews
ᅟHamilton, 2013, UK, primary care [19]	L	Hamilton 2005 [22]	GP usage and outcomes from practices and local trusts; qualitative interviews	Not reported	Risk assessment tools (RATs) supplied to 614 GPs at 164 practices for 6 months; interviews with GP cancer network leads and sample of GP users from practices with differing patient demographics.		Number of 2WW referrals and colonoscopies for patients >40; symptoms used in RATs; qualitative interview data.	RATs performed on patients <40; RATs that did not identify the reported symptoms.	1433	23 GP responders

*CRC* colorectal cancer, *ONS* office of national statistics, *FOBT* faecal occult blood test, *IBD* Inflammatory bowel disease, *Hb* haemoglobin, *WNS* Weighted Numerical Score developed by Selvachandran 2002 [30], *RAT* risk assessment tool

^a^Lung, gastro-oesophageal, pancreatic, renal tract, haematological, breast, ovarian, uterine, cervical and other cancer

^b^Lung, gastro-oesophageal, pancreatic, renal tract, haematological, prostate, testicular, and other cancer

^c^Adenoma with significant (> 25 %) villous features, or high grade dysplasia, including carcinoma-in-situ, or size 10 mm or larger

^d^Adenoma of any size or histology

^e^Polyp 10 mm or larger, or a polyp of any size with a villous histology

^f^rectal bleeding, weight loss, abdominal pain, loss of appetite

^g^2WW - Two week wait

^h^The method of developing the WNS is copyrighted and incompletely reported

### Risk model development and validation

Table 1 also summarizes the methods used to develop and validate the models. Of the 15 included models, nine were developed in primary care populations [21–27] and six in secondary care [20, 28–32]. Most (*n* = 11) had CRC as a single outcome, whilst two in secondary care predicted CRC combined with advanced adenoma (a polyp measuring 10 mm or bigger, or a polyp of any size with a villous histology [20]) or pre-malignant adenomas [28]. The remaining two reported CRC risk alongside the risk of cancers of several other sites [26, 27].

Most models were developed from either cross sectional (*n* = 6), prospective cohort (*n* = 4) or case-control studies (*n* = 4), with one developed based on clinical experience [30]. Four used self-administered questionnaires or interviews conducted by non-medically trained staff to gather information. The remaining 11 models required the input of a healthcare worker. Nine of the 15 models have been validated: one using bootstrap resampling [32]; two using a random split-sample [26, 27]; and six in external populations [21, 24, 25, 30]. Details of the methods and study populations for the validation studies are also given in Table 1.

### Variables included in the risk models

We categorized risk factor variables into five types: demographic, personal and family medical history, symptoms, signs, and investigations (Table 2). Seventeen variables were included in three or more models: four demographic variables (age, sex, smoking, alcohol); family history of CRC; eight symptoms (rectal bleeding, change in bowel habit, diarrhoea, constipation, abdominal pain, weight loss, loss of appetite, mucous in the stool); abnormal rectal examination; and three investigations (haemoglobin, mean cell volume, faecal occult blood testing). All models included symptoms, four included only symptoms [22–24], and most also included age (*n* = 11) and sex (*n* = 9). The choice of variable in each model was often influenced by the study design. For example, the Hamilton et al. 2005 risk model [22] and the CAPER score [24] were developed from case-control studies using primary care records and include symptoms plus additional signs and investigations without any demographic information or personal or family medical history. In contrast, the models developed by Adelstein [31, 32] from patient-completed questionnaires include symptoms plus demographic information and personal or family medical history.

**Table 2:** Variables included in risk models and TRIPOD classification of studies examining model performance

Author, year	TRIPOD level^a^	Demographic variables					Personal and Family Medical History		Symptoms									Signs		Investigations			
		Age	Sex	Smoking	Alcohol	Other	Family history of GI cancer	Other	Rectal bleeding	Change in bowel habit	Diarrhoea	Constipation	Abdominal pain	Weight loss	Loss of appetite	Mucous	Other	Abnor mal rectal examination	Other	Haemoglobin^b^	MCV	FOBT	Other
Models predicting gastrointestinal cancers and neoplasms																							
Adelstein, 2010 [32]	1b	●	●					Colonoscopy in last 10 years; history of diverticular disease, NSAID use, or aspirin use.	●				●			●	Anaemia^b^.						
Adelstein, 2011 [31]	1a	●	●	●		Education level.		Colonoscopy in last 10 years; history of colorectal polyps, IBS, NSAID use or aspirin use.	●							●	Anaemia^b^; fatigue.						
Fijten, 1995 [21]	1a, 4	●							●^d^	●													
Hamilton, 2005 [22]	1a								●		●		●	●				●	Abdominal tenderness	●		●	Blood glucose
Hamilton, 2009 [23]	1a								●	●	●	●	●	●						●	●		
Hippisley-Cox, 2012 (Male) [25]	2a, 4	●	N/A		●		●		●	●			●	●	●					●			
Hippisley-Cox, 2012 (Female) [25]	2a, 4	●	N/A				●		●				●	●	●					●			
Hurst, 2007 [28]	1a	●	●	●									●	●									sMMP-9
Lam, 2002 [20]	1a	●							●^e^							●							
Mahadavan, 2011 [29]	1a	●	●						●												●	●	eDNA; CEA
Marshall, 2011 (BB equation) [24]	3								●	●	●^f^	●^f^	●^f^	●				●	Abdominal mass	●	●	●	
Marshall, 2011 (CAPER score) [24]	4										●	●	●	●				●	Abdominal mass			●	
Selvachandran, 2002 (WNS) [30] ^c^	4, 4, 4, 4	●	●					‘Family history’, ‘relevant medical history’.	●	●				●	●		Tenesmus; urgency; incomplete emptying; perianal symptoms; ‘abdominal symptoms’; tiredness.						
Models predicting cancers of multiple organ systems alongside colorectal cancer																							
Hippisley-Cox, 2013 (Male) [27]	2a	●	N/A	●	●	BMI; Townsend deprivation score.	●	History of chronic pancreatitis, type 2 diabetes, or COPD; family history of prostate cancer.	●	●		●	●	●	●		Abdominal distension; heartburn; indigestion; dysphagia, haematemesis; haematuria; haemoptysis; neck lump; Night sweats; testicular lump; testicular pain; first occurrence of a venous thromboembolism; bruising; cough; impotence; nocturia; urinary frequency; urinary retention.			●			
Hippisley-Cox, 2013 (Female) [26]	2a	●	N/A	●	●	BMI; Townsend deprivation score.	●	History of chronic pancreatitis, type 2 diabetes, COPD, or endometrial hyperplasia/polyps; family history of breast cancer or ovarian cancer.	●	●		●	●	●	●		Abdominal distension; heartburn; indigestion; dysphagia; haematemesis; rectal bleeding; haematuria; haemoptysis; neck lump; weight loss; night sweats; breast lump; breast pain; nipple discharge or breast skin changes; inter-menstrual bleeding; post-menopausal bleeding; post-coital bleeding; first occurrence of a venous thromboembolism; bruising; cough.			●			

^a^Types of prediction model studies for each model defined according to the TRIPOD [17] guidelines. 1a – Development only; 1b – Development and validation using resampling; 2a – Random split-sample development and validation; 2b – Nonrandom split-sample development and validation; 3 – Development and validation using separate data; 4 – Validation study

^b^If anaemia was defined by a haemoglobin value, it was considered an investigation. Self-report of anaemia in the absence of a blood test was considered a symptom

^c^Selvachandran 2002 [30] describes a copyrighted model, the Weighted Numerical Score (WNS) that is incompletely reported

^d^specifically blood mixed with/on stool

^e^specifically rectal bleeding independent of straining or defaecation, blood clots, or dark red blood

^f^in addition to documentation of these symptoms, prescriptions used as a proxy. Laxative prescriptions taken to indicate constipation, anti-diarrhoeal prescriptions diarrhoea, and antispasmodic prescriptions abdominal pain

### Performance of risk models

#### Accuracy

**Fig. 2:**
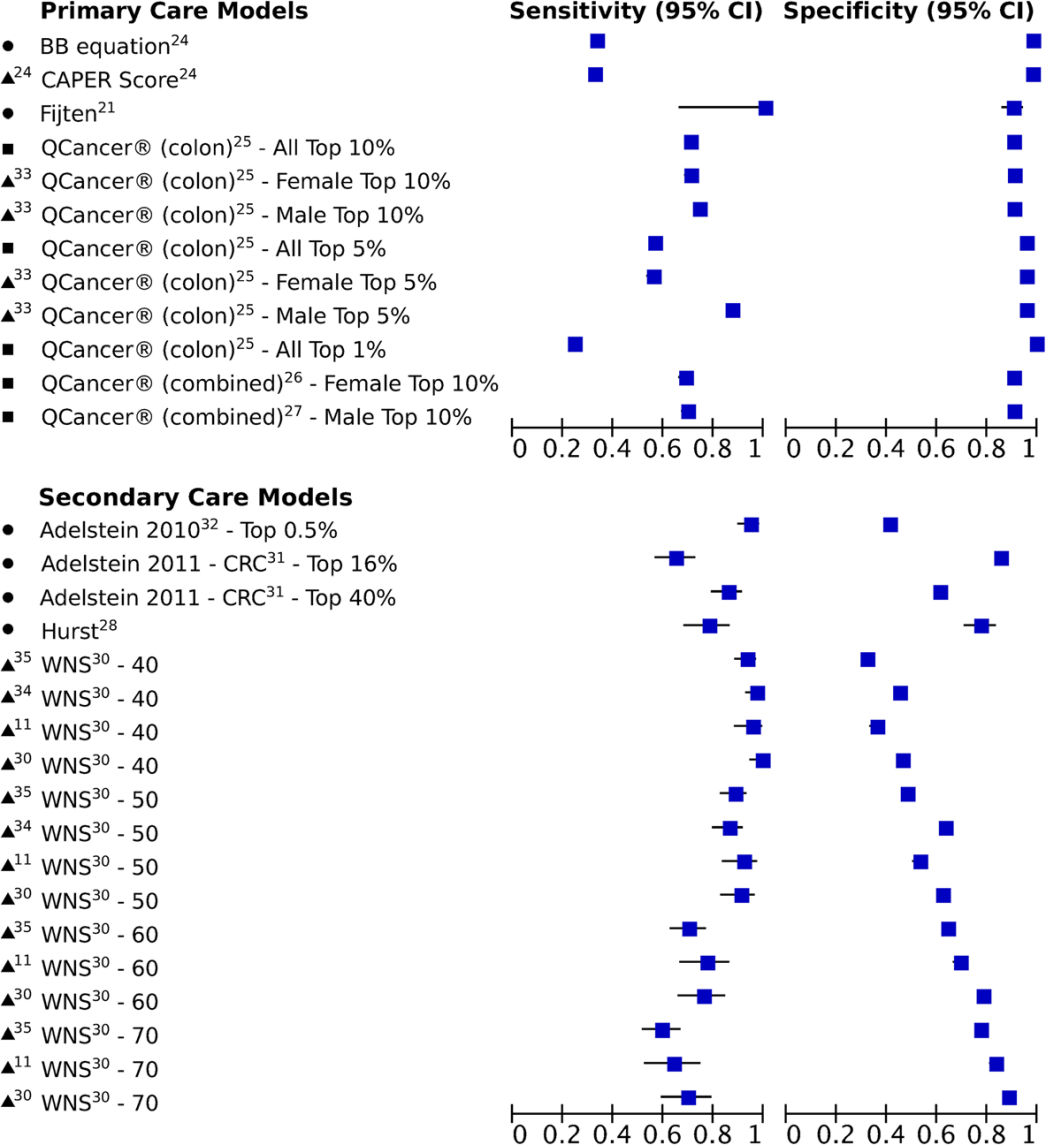
Sensitivities and specificities of risk prediction models at reported thresholds. ● indicates performance in a development population, ■ in an internal validation, and *black triangle* in an external validation (referenced). CRC – colorectal cancer. WNS – weighted numerical score

Sensitivity and specificity were reported for 11 of the 15 models in either development populations (*n* = 5), in random split-sample internal validation (*n* = 2), random split-sample internal validation and external populations (*n* = 2) or in external populations (*n* = 2); for four models these were provided for multiple thresholds. These values are summarized in Fig. 2 in which models are divided into those developed (and validated) in primary care, and those developed (and validated) in secondary care.

At all selected risk thresholds the seven models developed in primary care populations achieved high specificity (range 0.90–0.98), whilst the sensitivity ranged from 0.25 for the QCancer® (colon) model [25] with the threshold set at the top 1 % in internal validation, to 1.00 for the Fijten et al. model developed from a cohort of patients presenting to primary care practices in the Netherlands with rectal bleeding that contained only nine CRC cases [21]. Information on sensitivity and specificity of three models developed in primary care was reported in external cohorts: the CAPER score [24] and the two QCancer® (colon) models for male and female individuals [33]. Whilst all three models had similarly high specificity, the QCancer® (colon) models had higher sensitivity at all thresholds used for external validation and performed better in males then females.

Only one model developed in secondary care, the Weighted numerical scoring system (WNS) [30], has been externally validated. Four studies reported the sensitivity and specificity at four different thresholds (a score of 40, 50, 60 or 70). As expected, the lowest threshold of 40 had the highest pooled sensitivity (0.96 (95 % C.I. 0.93–0.97), *n* = 4 studies) and the lowest pooled specificity (0.40 (95 % C.I. 0.39–0.41), *n* = 4 studies), whilst the threshold of 70 had values comparable to those in primary care with a lower sensitivity of 0.64 (95 % C.I. 0.53–0.74) (*n* = 3) and higher specificity of 0.82 (95 % C.I. 0.81–0.83) (*n* = 3).

#### Discrimination

**Fig. 3:**
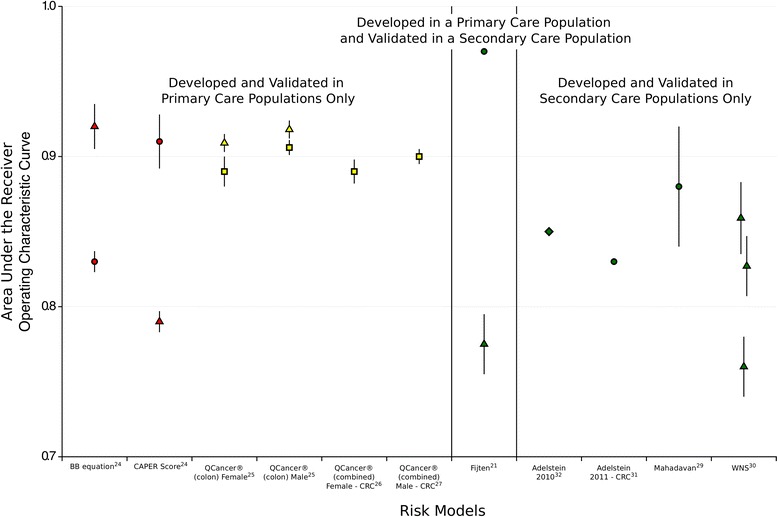
Area Under the Receiver Operating Characteristic curve of risk prediction models. ● indicates performance in a development population, *black diamond* in bootstrap resampling, ■ in an internal validation, and *black triangle* in an external validation (referenced). Point colours correspond to study design; *red* represents case-control, *green* cross-sectional and *yellow* cohort studies. CRC– colorectal cancer. WNS – weighted numerical score

The discriminatory performance of 11 of the 16 models was reported as the AUROC. As shown in Fig. 3, these range from 0.83 to 0.97 in model development populations, 0.89 to 0.91 in internal validation studies, and 0.76 to 0.92 in external validation studies. The highest discriminatory performance (AUROC 0.97) was achieved by the model developed by Fijten et al. in patients presenting to primary care practices in the Netherlands with rectal bleeding [21]. However, in an external validation study of the model in secondary care the discrimination fell to 0.78 [34]. The models demonstrating the best discrimination in external validation studies were the BB equation developed by Marshall et al. using a case-control design in the The Health Improvement Network (THIN) database of English primary care records [24] and the QCancer® (colon) male and female models developed from a cohort within the QResearch database of English primary care records [25].

#### Other performance measures and utility

Three models reported performance as positive predictive values (PPVs): the model by Lam et al. with a combined outcome of CRC or a polyp measuring 1 cm or bigger or any size with a villous histology derived in Hong Kong from a secondary care population in which PPVs ranged from 4.5 to 33.6 % [20], and the models developed by Hamilton et al. in the UK from primary care populations with PPVs ranging from 0.42 to 11 % [22], and from 0.04 to 4.5 % [23].

No studies reported numerical measures of calibration but Hippisley-Cox et al. 2012 showed plots of observed and predicted risk for the male and female QCancer® (colon) models in internal validation and these show overall good calibration [25].

Only one study assessed the utility of a risk model in practice: the risk score developed by Hamilton et al. 2009 [23] was assessed alongside a risk score for lung cancer in 165 UK general practices [19]. Paper, mouse-mat and desktop easel forms displaying the risk models were provided for a six month period. During this time there was an increase in cancer diagnostic activity, urgent referrals and cancer diagnoses when compared with the previous six months but as it was not a trial it is not possible to say whether these changes were due to the use of the risk model.

#### Comparison with existing guidelines

Although the aim of this review was not to assess the performance of referral guidelines for CRC, five papers [11, 24, 30, 34, 35] simultaneously compared the performance of risk models with that of published guidelines.

**Fig. 4:**
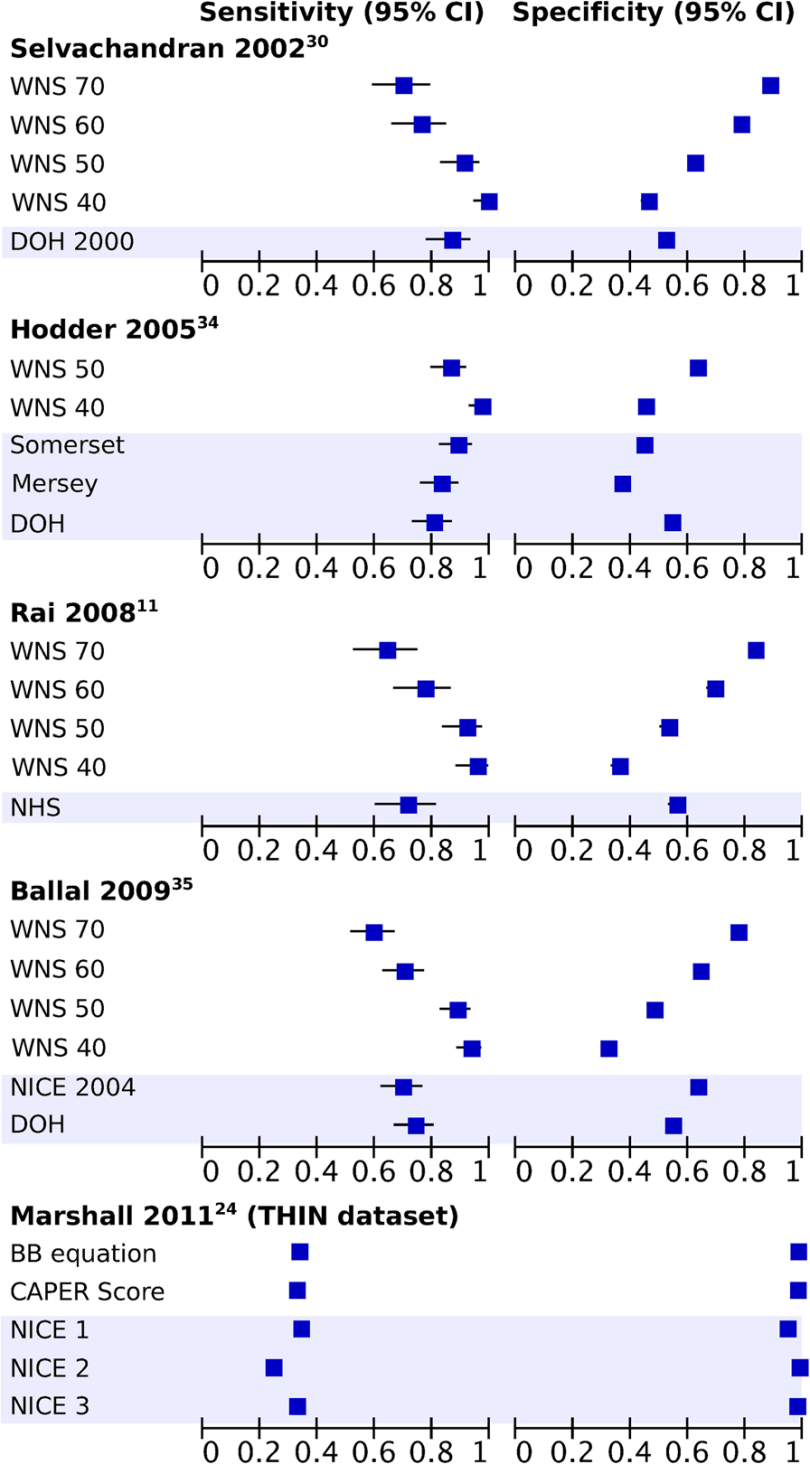
Sensitivities and specificities of risk prediction models and guidelines within the same population. Highlighted boxes indicate guidelines. In NICE 1, 2 and 3 a score of 1 was given for fulfilling any of the NICE high-risk criteria and a score of 100 for a positive faecal occult blood test (FOBT), abnormal rectal examination or abdominal mass. For NICE 1 a single consultation with diarrhoea or change in bowel habit (CIBH) was assumed to indicate a 6 week change. For NICE 2 two consultations for diarrhoea between 35 and 119 days apart were taken to indicate a change in bowel habit for > 6 weeks (CIBH coding not used). For NICE 3 two consultations for diarrhoea between 35 and 119 days apart, or a single consultation with CIBH, were taken to indicate a change in bowel habit for > 6 weeks. WNS – Weighted Numerical Score developed by Selvachandran et al. 2002 with cut-offs of 40, 50, 60 or 70. DOH – Department of Health

Four compared the sensitivity and specificity of the WNS to UK national or regional guidelines (Fig. 4): in three [11, 30, 34] a threshold of 50 or 60 in the WNS had a higher sensitivity and specificity than the guidelines, and in the fourth [35] a threshold of 60 had similar performance to both the NICE consultation guidelines published in 2004 [36] and the 2000 Department of Health [37] guidelines.

Figure 4 also shows Marshall et al.’s [24] study which compared the BB equation and CAPER score to three variations of NICE guidance in the UK THIN primary care database. They found the sensitivity and specificity of both risk models to be similar to the NICE 2005 guidelines [38]; however the authors also report the discrimination and show that both models outperformed the NICE guidelines with AUROCs of 0.83 (BB Equation) and 0.79 (CAPER Score), compared to 0.65 for the best performing interpretation of NICE guidelines. The same study [24] also compared the discrimination of the BB equation, CAPER score and NICE guidelines in a primary care case-control study with participants recruited from 21 practices in the UK. Again, the AUROCs achieved by the BB Equation (0.92) and CAPER Score (0.91) were significantly higher than the best performing interpretation of NICE guidelines (0.76).

## Discussion

### Strengths and weaknesses

The main strengths of this review are the broad search strategy and the systematic approaches used to identify studies and extract data. However, as with all systematic reviews, our conclusions are limited by the quality of published research. The included studies were heterogeneous in design, setting and duration of follow-up. Additionally, although we only included risk models with published performance data, two only provided positive predictive values, only six have been validated in external populations, and only one has been assessed for clinical utility or impact. This tendency for research into risk prediction tools to focus on model development rather than validation and impact is well documented [39, 40]. Nevertheless, it limits the conclusions that can be made about the potential role of these risk models in clinical practice.

### Comparison between risk models

Where data on discrimination and accuracy was reported there is little to distinguish between the models developed and validated in primary care. The four QCancer® models [25–27] were developed and validated using prospective cohort designs within two large UK primary care record databases, QResearch (development) and THIN (validation). All contain a combination of demographic, symptom, and investigation variables routinely recorded in electronic medical records, and all have AUROCs above 0.89 in either split-sample or external validation, and sensitivities around 0.7 with specificities over 0.95. The BB equation was also developed in the THIN database but using a case-control design [24] and has been validated in a dataset of paper-based primary care records from 21 English primary care practices, whilst the CAPER score was developed from those same primary care records and validated in the THIN database [24]. Both the BB equation and CAPER score performed better in the primary care record dataset, achieving comparable AUROCs and sensitivity and specificity to the QCancer® models. As Marshall et al. [24] describe, reasons for this may include the fact that clinical features of colorectal cancer were identified from both paper and electronic records and included analysis of free text in the primary care record dataset. The use of case-control designs instead of cohort studies for both the development and validation of these risk models, however, means that these measures may not accurately reflect their performance in population based cohorts due to the wide dispersion of risk factors in the cases and controls and the restricted distribution of matched variables.

Additionally none of these models have been validated outside data routinely collected by General practitioners (GPs). It is known that some symptoms are more likely to be recorded by a GP in patients in whom cancer is suspected. For example, patients coded as having a change in bowel habit are at greater risk than those with diarrhoea or constipation [41]. As a result of this coding bias it is likely that the recorded symptoms used in these models overestimate the significance of those symptoms in the presenting population. Whilst all these models in primary care can therefore accurately discriminate between patients in whom GPs have or have not chosen to routinely record these symptoms and could be used to identify those in whom further investigation or referral is necessary, how they perform in the consultation setting when GPs are having to decide whether the patient in front of them does or does not have a given symptom is not known.

Models developed and validated in secondary care settings were instead all based on cross-sectional studies of patients referred with symptoms of CRC with data collected using patient and/or physician questionnaires at the time of investigation. All have similar discrimination (AUROC 0.8 to 0.9) in development populations, but the only model to be externally validated is the WNS developed by Selvachandran et al. [30]. This has been validated in four separate populations with a prevalence of CRC of around 5 %. The AUROCs range from 0.76 [35] to 0.86 [30] and sensitivity and specificity from 0.96 and 0.40 to 0.64 and 0.82. A low threshold, with a high sensitivity, could therefore be used to identify those in whom further investigation is not required. However, all the patients included in these studies had already been assessed as high risk by primary care physicians so the score would be likely to perform less well in an un-referred primary care population, therefore validation in that setting is required.

### Implications for clinicians and policy makers

The risk models identified in this review have the potential to improve the diagnosis of CRC by helping clinicians to identify those patients presenting with symptoms of possible CRC in whom further investigation and referral is most appropriate. The potential advantages of risk prediction models in this context are that they can include combinations of symptoms and other risk factors, and different thresholds for action can be used. For example, a threshold with high sensitivity and high specificity could be used to define a high risk patients that require urgent referral, whilst one with very high sensitivity and low specificity could be used to identify those who do not require further investigation at that time.

Sackett and Haynes [42] identified four questions which must be addressed before incorporating diagnostic tests into clinical practice, however. The first three are concerned with test performance: whether test results are different between those with and without the condition; whether patients with certain test results are more likely to have the target disorder; and whether the test results distinguish patients with and without the target disorder. This review shows that risk models for CRC do exist which meet these criteria, with the best performing having sensitivities above 0.7, specificities above 0.9 and AUROCs over 0.9 in external validation studies.

Most contain variables that are easily obtainable in a single consultation and so could relatively easily be incorporated into practice. Whether any of them are any better than a clinician’s assessment is, however, uncertain. In the only study to compare a risk model with clinical judgement [30] the WNS was compared to the specialist clinical assessment of a comprehensive questionnaire-gathered history and there was no significant difference in discrimination. There is more evidence to suggest that the models are better than previous referral guidelines. Although not the primary aim of this review, in all cases where models were compared with guidelines the predictive models showed better discrimination and equal or better accuracy [11, 24, 30, 34, 35].

### Unanswered questions and future research

This review also cannot answer Sackett and Hayne’s fourth question - whether patients undergoing the diagnostic test fare better than similar untested patients. No studies have sought to address that. Before incorporating any of these risk models into practice, further research is therefore needed to validate the most promising models in clinical settings in comparison to clinical judgement and current referral guidelines, and to assess the impact of the use of these risk models in practice. Further work is also needed to consider whether CRC alone or in combination with advanced colorectal neoplasia or adenoma is the most appropriate outcome. This review focused on risk prediction models for CRC and only two models, which both reported only limited performance data and have not been validated, included advanced colorectal neoplasia or adenoma in addition to CRC [20, 28]. It is, therefore, not possible from this review alone to know how the performance of models predicting the combined outcome of advanced colorectal neoplasia and CRC compares to those with CRC as a single outcome. One study, however, reported the performance of risk models for CRC, advanced adenoma, or adenomas 6–9 mm in diameter separately within the same population [31]. The discrimination for the CRC model was substantially better (AUROC 0.87 compared to 0.70 and 0.67 for advanced adenoma and adenomas 6–9 mm in diameter respectively). This probably reflects the fact that many adenomas are asymptomatic and so identified less well by risk prediction models developed in symptomatic populations. The discriminatory performance for advanced adenoma in this symptomatic population is comparable with risk models developed for asymptomatic individuals [43]. This suggests that models with a combined outcome of advanced colorectal neoplasia and CRC may identify those with CRC less well than models with CRC as a single outcome. However, it is widely accepted that CRC arises from the adenoma-carcinoma sequence and so identification of patients with advanced colorectal neoplasia has the potential to reduce future incidence of invasive CRC. The choice of outcome(s) therefore depends on the purpose for which the risk models are to be used. If the priority is identification of patients with prevalent CRC, then a risk model including CRC as the sole outcome is likely to have the greatest discrimination and accuracy and allow targeting of referrals and further investigations most effectively. If the priority is instead to identify both prevalent CRC and those patients at high risk of developing CRC in the future, then a risk model including advanced colorectal neoplasia would be more appropriate.

The introduction of a two-step process into the recently updated NICE referral guidelines [44], in which the referral decision for individuals at intermediate risk is made based on the result of testing for occult blood in faeces, also provides an opportunity for research into incorporating other pre-referral tests into risk models. These include faecal immunochemical tests [16] and potentially more specialised tests, such as exfoliated DNA and carcinoembryonic antigen (CEA), which were of predictive value in secondary care developed models.

From work in other disease areas [45–49] we know that uncertainty about how to account for risk factors perceived to be important but not included in the tools, and the perception that clinical judgement is as good as or better than risk tools, contribute to the low uptake of risk models. Practical issues such as lack of time, poor knowledge or understanding of the tools, and poor computer software also restrict model use. Additionally, a recent study using simulated consultations with risk prediction tools for cancer has shown that clinicians may interpret symptoms inconsistently, leading to inaccurate and unreliable cancer risk assessment, and GPs were reluctant to use the tools for fear of alarming their patients if the risk information is presented too explicitly [50]. Research is therefore also needed to understand how best to incorporate risk prediction models into routine practice, including communication of risk information to patients, and to address the barriers to their use.

## Conclusions

To our knowledge this is the first systematic review of risk prediction models for CRC in symptomatic populations. We have shown that 15 models have been developed across both primary and secondary care populations. Many of these have good discrimination (AUROC > 0.85) and most contain variables that are easily obtainable in a single consultation. However, only six have been validated in external populations, and only one model has been assessed for clinical utility in a single before and after study with no control group. Further research is therefore needed before they can be incorporated into routine clinical practice.

## Abbreviations

2WW, Two-week wait referral system; AUROC, Area under the receiver operating characteristic curve; BB equation, The Bristol-Birmingham equation; CEA, Carcinoembryonic antigen; CIBH, Change in bowel habit; CRC, Colorectal cancer; DOH, Department of Health; FOBT, Faecal occult blood test; GP, General practitioner; Hb, Haemoglobin; IBD, Inflammatory bowel disease; NICE, National Institute for Clinical Excellence; ONS, Office of National Statistics; PPV, Positive predictive value; THIN, The Health Improvement Network; WNS, Weighted numerical scoring system

## Additional files

Additional file 1: PRISMA checklist, Completed PRISMA checklist (DOC 116 kb) Additional file 2: Search strategies, Full details of search strategies used for Medline and EMBASE. (DOCX 24 kb)
