# Supplementary material for: Risk prediction models for colorectal cancer in people with symptoms: a systematic review
Source: BMC Gastroenterol. 2016 Jun 13;16:63. doi: 10.1186/s12876-016-0475-7 (PMC4907012; doi:10.1186/s12876-016-0475-7)
Supplement: Additional file 2: — Search strategies, Full details of search strategies used for Medline and EMBASE. (DOCX 24 kb) [file 12876_2016_475_MOESM2_ESM.docx]

**Additional file 2 – Search Strategies**

The specific strategies for Medline and EMBASE were:

**Medline**

exp Risk Factors/ or risk*.mp. or exp Risk/ or exp Risk Assessment/

2 chance*.mp.

3 likelihood*.mp. or exp Probability/

4 1 or 2 or 3

5 predict*.mp.

6 exp "Early Detection of Cancer"/

7 exp models, statistical/

8 exp Models, Statistical/ or model*.mp.

9 Score*.mp.

10 5 or 6 or 7 or 8 or 9

11 exp Colorectal Neoplasms/

12 ((colorectal or rect* or colon*) adj3 (cancer* or neoplas* or tumour* or tumor*)).ti,ab.

13 11 or 12

14 4 and 10 and 13

15 Review/

16 Comment/

17 Letter/

18 Editorial/

19 15 or 16 or 17 or 18

20 14 not 19

21 limit 20 to (humans and yr="2000 -Current")

**Embase**

1 exp cancer risk/ or risk*.mp. or exp risk/ or exp risk factor/ or exp risk assessment/

2 chance*.mp.

3 exp probability/ or likelihood*.mp.

4 1 or 2 or 3

5 exp mathematical model/ or model*.mp. or exp model/

6 exp prediction/ or predict*.mp.

7 score.mp.

8 5 or 6 or 7

9 ((colorectal or rect* or colon*) adj3 (cancer* or neoplas* or tumour* or tumor*)).ti,ab.

10 exp colon tumor/

11 exp rectum tumor/

12 exp colorectal cancer/

13 9 or 10 or 11 or 12

14 4 and 8 and 13

15 review.pt.

16 letter.pt.

17 editorial.pt.

18 15 or 16 or 17

19 14 not 18

20 limit 19 to (human and yr="2000 -Current")
